# Janus Kinase Inhibitors in the Treatment of Vitiligo: A Review

**DOI:** 10.3389/fimmu.2021.790125

**Published:** 2021-11-18

**Authors:** Fei Qi, Fang Liu, Ling Gao

**Affiliations:** ^1^ Department of Dermatology, Beijing Chaoyang Hospital, Capital Medical University, Beijing, China; ^2^ China CDC Key Laboratory of Radiological Protection and Nuclear Emergency, National Institute for Radiological Protection, China Centers for Disease Control, Beijing, China

**Keywords:** vitiligo, JAK/STAT-1 signaling pathway, JAK inhibitors, chemokines, IFN - interferon

## Abstract

Vitiligo is a multifactorial reversible skin disorder characterized by distinct white patches that result from melanocyte destruction. Activated CXCR3^+^ CD8^+^ T cells promote melanocyte detachment and apoptosis through interferon-gamma (IFN-γ secretion and chemokines secreted by keratinocytes through the Janus kinase (JAK)/signal transducer and activator of transcription (STAT)-1 signaling pathway results in further recruitment of CXCR3^+^ CD8^+^ T cells and the formation of a positive-feedback loop. JAK inhibitors target the JAK/STAT pathway and are now approved to treat many immune-related diseases. In the treatment of vitiligo, JAK inhibitors, including ruxolitinib, baricitinib, and tofacitinib, are effective, supporting the implication of the IFN-γ-chemokine signaling axis in the pathogenesis of vitiligo. However, more studies are required to determine the ideal dosage of JAK inhibitors for the treatment of vitiligo, and to identify other inflammatory pathways that may be implicated in the pathogenesis of this condition.

## Introduction

Vitiligo is an acquired, idiopathic autoimmune disorder characterized by patchy depigmentation in the skin, hair, or both (1). The disorder affects approximately 0.5–2% of adults and children globally and presents with amelanotic, milky white, and well-demarcated macules or patches surrounded by normal skin (2). Patients with vitiligo, especially those with darker skin tones, can suffer from stigmatization, negatively impacting their mental health and quality of life (3–5). Traditional therapeutic methods include systemic glucocorticoids and phototherapy. Although new therapeutic methods have been tested in clinical trials, universally effective treatment for vitiligo remains elusive because of the incomplete understanding of its pathogenesis (1, 6–8).

## The Interferon-Gamma-Chemokine Axis in the Pathogenesis of Vitiligo

Depigmentation that characterizes vitiligo is caused by progressive melanocyte destruction (8). Both *in vivo* and *ex vivo* studies have provided strong evidence that melanocyte-specific CD8^+^ T cells predominantly infiltrate the dermal-epidermal junction adjacent to melanocytes in the border of depigmented lesions and participate in the elimination and destruction of melanocytes (9–12).

IFN-γ the key cytokine produced by CD8^+^ T cells, plays a central role in the pathogenesis of the disease (13). The expression of IFN-γ-induced genes including the T cell chemokine receptor (CXCR3) and its multiple ligands, CXCL9, CXCL10, and CXCL11, is upregulated in depigmented skin lesions. The expression of IFN-γ-induced genes is consistent with other findings: enriched infiltration of CXCR3^+^ CD8^+^ T cells, including melanocyte-specific CD8^+^ T cells, found in biopsies of vitiligo lesions, and increased CXCR3 receptor expression on melanocyte-specific T cells in the blood and skin of patients with vitiligo (14–18).

**Figure 1:**
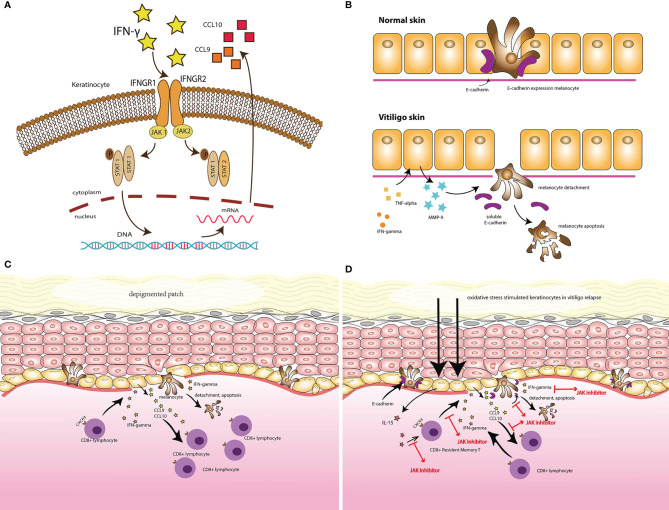
**(A)** IFN- γ signaling and the Janus kinase (JAK)/signal transducer and activator of transcription (STAT) pathway in vitiligo. **(B)** The secretion of MMP-9 by keratinocytes, in response to IFN-γ and TNF-α, induced melanocytes detachment through E-cadherin disruption and released its soluble form, in the vitiligo skin comparing with the normal skin. **(C)** Illustrates the vitiligo pathogenesis: the IFN-γ-chemokine axis, with its associated positive-feedback loop: the autoreactive CD8+ T cells produce IFN-γ, which promotes depigmentation; IFN-γ simultaneously stimulates keratinocytes to express CXCR3 that binds to CXCL9 to recruit more melanocyte-reactive T cells. In addition, CXCL10 recruits T cells within the skin through the CXCR3 receptor, which prolongs and exacerbates the established vitiligo lesion. **(D)** The potential target for JAK inhibitors to treat vitiligo.

Based on multiple studies conducted in mouse vitiligo models, the IFN-γ-chemokine axis, with its associated positive feedback loop, has been identified as a potential pathway in the initiation and progression of vitiligo. Autoreactive CD8^+^ T cells produce IFN-γ, which promotes depigmentation. IFN-γ simultaneously stimulates keratinocytes to express CXCR3, which binds to CXCL9 to recruit more melanocyte-reactive T cells. In addition, CXCL10 recruits T cells within the skin through the CXCR3 receptor, which prolongs and exacerbates the established vitiligo lesion (**Figure 1C**) (15, 19–21).

## IFN-γ Signaling and the Janus Kinase/Signal Transducer and Activator of Transcription Pathway In Vitiligo

Janus kinases (JAKs) are a family of cytoplasmic tyrosine kinases (TYKs) with a structure consisting of four domains (22). They aid cytokine-mediated signal transduction through the JAK/STAT pathway (23). The key members of this unique tyrosine kinase family include JAK1, JAK2, JAK3, and TYK2 (24). IFNs stimulate the JAK/STAT pathway, leading to the expression of IFN-stimulated genes (ISGs) (25). IFN-γ, a type II interferon, activates the JAK/STAT1 pathway by binding to a cell-surface receptor composed of two subunits, IFNGR1 and IFNGR2, related to JAK1 and JAK2, respectively. This leads to the phosphorylation of STAT1, which initiates gene transcription (26). Given the role of JAK1 and JAK2 in the JAK/STAT pathway, IFN-γ signals can also be blocked by inhibiting JAK1 or JAK2. The JAK/STAT pathway is involved in a variety of immune-related disorders, and inhibitors targeting JAKs are now used to treat numerous diseases (**Figure 1A**) (27).

Studies have demonstrated the central role of the JAK/STAT pathway in vitiligo. Case-control studies have demonstrated a stepwise pattern of increased expression of JAK1 and JAK3 in lesion-free, perilesional, and vitiliginous skin, respectively, and high expression of JAK 1 was also shown to decline after narrow-band ultraviolet B therapy (23, 28). When the JAK/STAT signaling pathway is inhibited, the detachment of low E-cadherin-expressing melanocytes in the basal layer of the epidermis, a critical step before melanocyte apoptosis, is disrupted. This has also been shown to decrease MMP-9, the key potent factor secreted by keratinocytes in response to IFN-γ and TNF-α, which was found increased in the skin and serum vitiligo patients and stimulated E-cadherin disruption (29, 30). Interleukin (IL)-15 is a cytokine with a special delivery mechanism that transmits signals through the JAK/STAT pathway with JAK1 and JAK3 stimulation, which results in STAT5 activation (31). In the pathogenesis of vitiligo relapse, oxidative stress stimulates keratinocytes *via* nuclear factor (NF)-*κ*B signaling to express IL-15 and IL-15 R*α*, which activate CD8^+^ resident memory T cells through the JAK/STAT signaling pathway (**Figure 1B**) (32).

## The Use of JAK Inhibitors in Vitiligo Treatment

In mouse vitiligo models, neutralization of IFN-γ antibodies prevent the accumulation of CD8^+^ T cells and lesion depigmentation. JAK inhibitors have been shown to block IFN-γ signaling, contributing to re-pigmentation in individuals with vitiligo. Tofacitinib (Pfizer, New York, NY, USA), ruxolitinib (Celgene, Summit, NJ, USA), and baricitinib (Indianapolis, IN, USA) are the three most commonly reported JAK inhibitors used in vitiligo treatment (**Figure 1D**) (**Tables 1A**, **B**).

**Table 1A:** Trials of emerging JAK inhibitors in vitiligo.

NCT number	Sponsor	Nationality	Trial phase	Treatment group	Drug type	Subject	Allocation	Status	Results	Side effect
NCT04896385	Incyte Corporation	The United States and Canada	2	Group1: ruxolitinib cream Group2: vehicle	JAK1/2 inhibitor	60	Randomized, double-blind	Recruiting	Not available	Not available
NCT02809976	Tufts Medical Center	The United States	2	Group 1: ruxolitinib 1.5% phosphate cream twice daily	JAK1/2 inhibitor	11	Single group, open-label	Completed	4 patients presented significant facial improvement, 23% of patients decreased VASI	Only mild side effects
NCT03099304	Incyte Corporation	The United Kingdom	2	Group 1: ruxolitinib cream 1.5% twice daily Group 2: ruxolitinib cream 1.5% once daily Group 3: ruxolitinib cream 0.5% once daily Group 4: ruxolitinib 0.15% once daily Group 5: vehicle	JAK1/2 inhibitor	157	Randomized, double-blind	Completed	More patients in cream 1.5% twice daily, 1.5% once daily, 0.5% once-daily groups achieved F-VASI50 than the control groups.	Only mild side effects
NCT04052425	Incyte Corporation	The United States	3	Group 1: ruxolitinib cream Group 2: vehicle	JAK1/2 inhibitor	330	Randomized, double-blind	Active	Not available	Not available
NCT04057573	Incyte Corporation	The United States	3	Group1: ruxolitinib cream Group 2: vehicle	JAK1/2 inhibitor	334	Randomized, double-blind	Active	Not available	Not available
NCT04530344	Incyte Corporation	The United States	3	Group 1: ruxolitinib cream Grorup2: vehicle	JAK1/2 inhibitor	500	Randomized, double-blind	Recruiting	Not available	Not available
NCT04822584	University Hospital, Bordeaux	France	2	Group 1: baricitinib Group 2: placebo	JAK1/2 inhibitor	48	Randomized, double-blind	Not yet recruiting	Not available	Not available
NCT04103060	Dermavant Sciences GmbH	The United States	2	Group 1: cerdulatinib, 0.37% gel, twice daily Group 2: vehicle gel, twice daily	SYK and JAK inhibitor (without JAK2)	33	Randomized, double-blind	Completed	Not available	Not available
NCT03715829	Pfizer	The United States	2	Group 1: PF-06651600 ritlecitinib Group 2: placebo Group 3: PF06700841 brepocitinib	Brepocitinib: TYK2/JAK1 inhibitor Ritlecitinib: JAK3/TEC inhibitor	366	Randomized, double-blind	Completed	Not available	Not available
NCT03468855	Aclaris Therapeutics, Inc.	The United States	2	Group: **ATI-50002** ifidancitinib twice daily	JAK1 and JAK3 inhibitor	34	Single group, open-label	Completed	Mean change in F-VASI: -0.067 (0.2411) VNS: 2.2 (0.66)	1 Acute myocardial infarction, 1 Alcoholic Pancreatitis

VASI, Vitiligo Area Scoring Index; F-VASI, Facial Vitiligo Area Scoring Index; VNS, Vitiligo Noticeability Scale.

**Table 1B:** Case report and cases series with JAK inhibitors in vitiligo.

	Authors	Nation	Treatment	Drug type	Patient	Presentation at baseline	Results	Side effect
Ruxolitinib	Harris et al. (19)	The United States	Ruxolitinib 20 mg orally twice daily	JAK1/2 inhibitor	1	Macular depigmentation on his face, trunk, and extremities	51% facial pigmentation compared to 0.8% at baseline	No side effects
Tofacitinib	Craiglow et al. (33)	The United States	Tofacitinib 5 mg every other day for a week, then 5 mg daily	JAK 1/3 inhibitor	1	White macules and patches involving the forehead, trunk, and extremities involving approximately 10% of body surface area	Approximately 5% of the total body surface area remained depigmented	No side effects
	Liu et al. (34)	The United States	Tofacitinib 5–10 mg daily or twice daily	JAK 1/3 inhibitor	10	8 generalized vitiligo, 2 acral involvement	5 patients who achieved some reversal of disease, re-pigmentation occurred only in sun-exposed areas of skin in 3 of them	No side effects
	Kim et al. (35)	The United States	Tofacitinib 5 mg twice daily, plus low-dose, narrow-band UV-B twice weekly	JAK 1/3 inhibitor	2	Patient 1: white patches involving about 75% of her face as well as white patches on the neck, chest, forearms, hands, and shins Patient 2: 90% of his face, as well as white patches on the torso and arms	Patient 1: nearly complete re-pigmentation of her face 75% or greater re-pigmentation of her neck, chest, forearms, shins, and only minimal freckling of the dorsal hand Patient 2: 75% facial re-pigmentation. No re-pigmentation occurred at the other body sites	No side effects
Tofacitinib	Mobasher et al. (36)	The United States	Topical use of 2% tofacitinib twice daily	JAK 1/3 inhibitor	16	–	13 >90% repigmentation; 5 25–75% repigmentation; 4 5–15(%) repigmentation; 2 no change	No side effects
Baricitinib	Mumford et al. (37)	Australia	Oral baricitinib 4 mg daily	JAK1/2 inhibitor	1	Vitiligo affecting the hands and forearms	Amost complete re-pigmentation of the hands and forearms was observed	No side effects

### Ruxolitinib

Ruxolitinib (INCB-018424) is a small-molecule inhibitor that selectively targets JAK1 and JAK2 (38). The oral form of ruxolitinib was first approved in 2011 to treat polycythemia vera, essential thrombocythemia, and myelofibrosis. Although oral ruxolitinib has been shown to improve skin conditions, such as alopecia areata (39, 40), topical administration of ruxolitinib resulted in higher concentrations in both the epidermis and dermis with minimal deleterious systemic effects versus oral administration, demonstrating sustained and near-complete blockage of the JAK/STAT signaling pathway in the tissues to which it was applied, with negligible plasma concentrations (41). Therefore, more studies have been conducted on ruxolitinib cream to investigate its efficacy in treating inflammatory skin disorders, such as alopecia areata, atopic dermatitis, lichen planus, and psoriasis (41, 42).

Rapid skin re-pigmentation was observed in male patients with vitiligo and alopecia areata treated with oral ruxolitinib, with marked declines in serum CXCL10 levels after administration, indicating that ruxolitinib’s mechanism of action may involve disruption of IFN-γ signaling and JAKs. The role of IFN-γ- and CD8+ T cell-dependent cytokine activity, which is implicated in the pathogenesis of alopecia areata, may thus also play a role in the pathogenesis of vitiligo (15, 43–45). Although the mechanism of action of ruxolitinib cream in the treatment of vitiligo is still unclear, studies on mice and human tissues have found that in addition to blocking IFN-γ and its downstream effector, JAKs, ruxolitinib also inhibited the differentiation and migration of human dendritic cells (DCs) *ex vivo.* This reduced DC-induced antigen-specific CD4^+^ and CD8^+^ T cell responses and the induction of CD8^+^ cytotoxic T cell responses, which are the key cell responses that are hypothesized to participate in the pathogenesis of vitiligo, *in vivo* (46). An ongoing phase 2 study (NCT04896385), sponsored by the Incyte Corporation, is currently in the recruitment stage. This study aimed to investigate the mechanism of action of ruxolitinib cream in treating patients with vitiligo by evaluating changes in immune biomarkers, including CXCL10 (47).

Based on preliminary findings in mice and human tissues, clinical trials with topical ruxolitinib have been conducted. In a 20-week, open-label, phase 2 trial (NCT02809976), 12 patients received topical 1.5% ruxolitinib cream applied to vitiligo lesions twice daily. Compared with baseline, four patients showed significant improvement in facial lesions, and all patients showed a 23% average decrease in the Vitiligo Area Scoring Index (VASI) (48). A 32-week extension study followed, but no improvement was found in skin lesions that were previously non-responsive to ruxolitinib. However, five patients followed up after the trial maintained their response to treatment for up to 6 months after treatment discontinuation (49).

In another double-blind phase 2 trial (NCT03099304), 157 adult patients with vitiligo from 26 hospitals in the United States were randomly assigned 1:1:1:1:1 to receive topical ruxolitinib cream 1.5% twice daily, 1.5% once daily, 0.5% once daily, 0.15% once daily, or a vehicle for 24 weeks, respectively. The percentage of patients who achieved more than 50% improvement from baseline in Facial VASI (F-VASI 50) at week 24 was set as the primary endpoint to evaluate treatment efficacy in each group. After the 24-week treatment period, significantly more patients in the groups receiving ruxolitinib cream 1.5% twice daily, 1.5% once daily, and 0.5% once daily achieved F-VASI50 than patients in the control groups. Patients who were assigned in the three positive responsive groups receiving ruxolitinib cream 1.5% twice daily, 1.5% once daily, and 0.5% once daily were asked to remain their original treatment dose up to 52 weeks. At week 52, patients in these three treatment groups showed substantial repigmentation of vitiligo lesions and good dose tolerance, indicating that topical ruxolitinib may be a good option for vitiligo management (50).

The goal of the two phase 2 studies was to assess the generalizability and investigate the efficacy of different concentrations and dosages of ruxolitinib cream (51). However, the average age of patients in both studies was >40 years, and only patients with non-segmental vitiligo were included. There were no studies specified to younger patients between 20 and 30 years of age (the most affected age group), and patients with vitiligo with darker skin (the patient group are more likely to be associated with concurrent autoimmune conditions) (52, 53). In both studies, enrolled patients’ total affected body surface area was under 10% on average, and the topical ruxolitinib exposure was under 3.74 g. Although two multi-center, double-blind, vehicle-controlled phase 3 studies (NCT04052425 and NCT04057573) and one 52-week long-term large sample phase 3 study (NCT04530344) are now being performed to evaluate the efficacy, safety, and the duration of response following ruxolitinib cream withdrawal, further evaluation of the use of ruxolitinib cream is needed to determine its safety and efficacy when used in patients with larger areas of vitiligo lesions (54–57).

### Tofacitinib

Tofacitinib is a selective JAK1 and JAK3 inhibitor approved to treat moderate and severe rheumatoid arthritis (43). Both oral and topical forms of tofacitinib have shown efficacy in treating immune-mediated skin disorders, including plaque psoriasis, atopic dermatitis, and alopecia areata (58–60). Oral administration of tofacitinib was first used in a female patient with vitiligo who had approximately 10% depigmentation in her total body surface area, which was unresponsive to the traditional application of topical corticosteroid ointment and tacrolimus ointment. Given the hypothesized common pathogenesis in alopecia areata and vitiligo, the patient was prescribed 5 mg of oral tofacitinib citrate on alternate days, which was increased to 5 mg daily from week 4. After 5 months of treatment, only 5% of the patient’s total body surface area remained depigmented. No side effects were reported during the treatment period (33).

In a retrospective study of 10 tofacitinib-treated patients with vitiligo, changes in their autoimmune responses were evaluated through suction blister sampling. Ten patients underwent tofacitinib therapy at a dosage of 5–10 mg daily or twice daily for an average of 9.9 months, with only half of the patients achieving re-pigmentation that occurred in sun-exposed areas or areas that received phototherapy only. Flow cytometry revealed a decline in the number of CD8^+^T cells after tofacitinib treatment, but there was no change in the percentage of melanocyte-specific T cells. In addition, chemokines, such as CXCL9 and CXCL10, were reduced and became undetectable after tofacitinib treatment (34). These findings indicate that re-pigmentation of vitiligo lesions may require both JAK inhibitors (to inhibit local inflammation) and light exposure (to stimulate melanocytes) (35), which is consistent with the finding that sun-exposed areas, such as the hands and face, are more responsive to topical ruxolitinib treatment (48).

Topical 2% tofacitinib cream was also administered to 16 patients with vitiligo, including 11 patients with generalized vitiligo. Consistent with previous studies, more significant responses were noted for facial lesions and patients with darker skin types, while no superior responses were observed in those who received concomitant phototherapy, which contrasts with previously reported results (36). There are no registered clinical trials currently being performed to investigate the use of tofacitinib in vitiligo treatment, and further research is necessary to determine its safety and efficacy, as well as the role of phototherapy in combination with tofacitinib.

### Baricitinib

Only one case report has described re-pigmentation in vitiligo lesions treated with baricitinib, a selective JAK1 and JAK2 inhibitor (43). A 67-year-old man with vitiligo involving the hands and forearms noticed complete re-pigmentation when he substituted his tofacitinib 5 mg twice daily with baricitinib 4 mg daily for the treatment of rheumatoid arthritis (37). A new phase 2 study (NCT04822584) is currently being conducted to evaluate the safety and efficacy of the combination of baricitinib and phototherapy in vitiligo treatment (61).

### Other JAK Inhibitors

A few registered ongoing trials are focusing on the use of second-generation JAK inhibitors in the treatment of patients with vitiligo, which may elucidate additional inflammatory pathways that may be important in the pathogenesis of vitiligo (13). Cerdulatinib (PRT062070) is a small molecule reversible inhibitor that targets both spleen tyrosine kinase (SYK) and JAK family members while sparing JAK2 (62, 63). A study (NCT04103060) was registered to assess the safety and tolerability of 0.37% cerudulatinib gel in treating adult patients with vitiligo (64).

Ritlecitinib (PF-06651600), a novel irreversible inhibitor of JAK3 and tyrosine kinase expressed in the hepatocellular carcinoma (TEC) kinase family, was previously used in the treatment of moderate-to-severe rheumatoid arthritis (65). Brepocitinib (PF-06700841) is an oral TYK2/JAK1 inhibitor used to treat moderate-to-severe plaque psoriasis (66). An ongoing registered trial (NCT03715829) carried out by Pfizer compared both ritlecitinib and brepocitinib in vitiligo treatment with/without the combination of phototherapy, which may offer additional insights into vitiligo pathogenesis (1, 67).

Ifidancitinib (ATI-50002) is another dual JAK1, and JAK3 inhibitor used to treat alopecia areata with oral and topical formulations (2, 68). The efficacy of a 0.46% ifidancitinib solution on non-segmental facial vitiligo was investigated through a phase 2, open-label study (NCT03468855). Of the 34 enrolled patients, 23 completed the 24-week treatment protocol with ifidancitinib Solution twice daily and improved F-VASI and the Vitiligo Noticeability Scale (VNS) (2, 69).

## Discussion

As more studies have investigated the pathogenesis of vitiligo over the past few decades and discovered more targeted, effective, and promising treatments. With the discovery of the role of the IFN-γ signaling axis in vitiligo, more clinical trials with JAK inhibitors have been performed, demonstrating remarkable efficacy in vitiligo management. However, there may be additional inflammatory pathways involved in the pathogenesis of vitiligo, which are yet to be elucidated. TYK2, another member of the JAK family, plays a ubiquitous role in signal transduction with type I interferon (IFN-*α*), which also induces the expression of CXCL9 and CXCL10 by keratinocytes in vitiligo (70). The common pathogeneses between vitiligo and other autoimmune diseases may also provide novel insights into vitiligo, such as the discovery of ruxolitinib and baricitinib in vitiligo treatment. Whether phototherapy is required to stimulate melanocyte regeneration in vitiligo treatment remains controversial, and future studies should be conducted to determine the optimal treatment regimen. Finally, current studies rarely focus on generalized vitiligo; hence, research should be conducted in patients who require topical JAK inhibitors to larger body surface areas. The differences in efficacy and safety between oral and tropical formulations of JAK inhibitors are another opportunity for future research.

## Author Contributions

FL and LG contributed to the conception and design of this study. FQ wrote the first draft of this manuscript. All authors contributed to manuscript revision, read, and approved the submitted version.

## Funding

This work was supported by Beijing Municipal Natural Science Foundation (7202139).

## Conflict of Interest

The authors declare that the research was conducted in the absence of any commercial or financial relationships that could be construed as a potential conflict of interest.

## Publisher’s Note

All claims expressed in this article are solely those of the authors and do not necessarily represent those of their affiliated organizations, or those of the publisher, the editors and the reviewers. Any product that may be evaluated in this article, or claim that may be made by its manufacturer, is not guaranteed or endorsed by the publisher.
